# Biofilms, mobile genetic elements and the persistence of pathogens on environmental surfaces in healthcare and food processing environments

**DOI:** 10.3389/fmicb.2024.1405428

**Published:** 2024-06-04

**Authors:** Carine Nkemngong, Peter Teska

**Affiliations:** ^1^AvantGuard Inc DBA Halomine Inc., Ithaca, NY, United States; ^2^Diversey-A Solenis Company, Fort Mill, SC, United States

**Keywords:** environmental biofilms, healthcare, mobile genetic elements, food processing, biofilms

## Abstract

Biofilms are the natural state for bacterial and fungal species. To achieve surface hygiene in commercial facilities, the presence of biofilms must be adequately considered. However, standard disinfectant and sanitizer efficacy tests required by the US-EPA and the European Committee for Standardization (CEN) do not currently consider the role of environmental biofilms. This selective review will discuss what biofilms are and why they are important. We will also cover where they are commonly found in healthcare and food processing facilities and explore how current antimicrobial test methods required for product registration do not test for the presence of biofilms. Additionally, we will explore how a lack of efficacy against biofilms may play a role in the development of antimicrobial resistance in healthcare facilities due to the exchange of mobile genetic elements that occur readily in a biofilm matrix.

## Introduction

Although unicellular, in nature, bacteria exist in aggregates referred to as biofilms. This community lifestyle allows unicellular microorganisms to enjoy the benefits of multicellular populations, such as being encased in a self-produced extracellular polymeric matrix (Stanley and Lazazzera, 2004). Additionally, microorganisms in biofilms are better suited to withstand environmental stress through the development of persister cells (Keren et al., 2004) and the production of extracellular DNA (eDNA) (Campoccia et al., 2021). eDNA facilitates lateral gene transfer; allowing for the exchange of virulence traits. These virulence traits may include an enhanced ability to adhere to hydrophobic surfaces, which may otherwise not be ideal for bacterial colonization and growth (Das et al., 2011). Moreover, chelation and the innate neutralization of positively charged antimicrobial peptides have been associated with eDNA secretion by biofilms. This suggests that the secretion of transferrable mobile genetic elements by bacterial biofilms may be triggered by the use of certain classes of antimicrobials such as peptides. However, this may be concentration dependent as daily use concentrations of antimicrobial actives for environmental surface disinfection are typically orders of magnitude higher than minimum biocidal concentrations; allowing for innate defense mechanisms that may allow for the transfer of intact eDNA to be impaired. Current scientific evidence (Potera, 2010; Das et al., 2011; Lax et al., 2017; Campoccia et al., 2021) suggests the advantages associated with a biofilm mode of life provide a fitness advantage and may explain their prevalence on a wide range of environmental surfaces. This review will focus on the prevalence of biofilms on environmental surfaces in healthcare and food processing environments, interactions between biofilm formers and non-biofilm formers and the role of mobile genetic elements in multi-species biofilms. Additionally, we will discuss how the prevalence of biofilms on environmental surfaces may impact hygiene and sanitation and vice versa. Briefly, we will also highlight regulatory differences between the US Environmental Protection Agency and the European Standards Committee for carrying biofilm claims on antimicrobial products.

## Biofilms and environmental surfaces in healthcare

In a patient room, where the patient is present in the same indoor space for days to weeks, little is known about how the room microbiome changes due to the presence of the patient. One study investigating this issue by Lax et al. found that there was a bidirectional exchange between the patient room and the patient’s skin and that these two communities became similar over a patient’s stay. The bedrail microbiota was the surface found to most rapidly resemble the patient’s skin, while the room floor had the strongest correlation with the patient’s skin microbiota over time (Lax et al., 2017). Changes in the skin microbiota were only weakly affected by the clinical therapies the patient received. Importantly, metagenomic analysis of samples from room surfaces found antibiotic resistant genes at higher levels than on the patient’s skin, suggesting the room surfaces could be reservoirs for microorganisms with virulent traits (Lax et al., 2017). Additionally, the likely transfer of pathogens from surfaces in patient rooms to human skin and vice versa highlights the importance of residual antimicrobial interventions with demonstrable log reductions in real world settings.

In a 2018 study, Ledwoch et al. sampled over 60 surfaces from UK hospitals and found multi-species bacterial biofilms on over 95% (57/60) of the surfaces (Ledwoch et al., 2018). The surfaces evaluated included food trolleys, hospital commodes, patient folders, computer keyboards and sanitizer bottles (Ledwoch et al., 2018). In another study, Costa et al. completed scanning electron microscopy (SEM) or confocal laser scanning microscopy (CLSM) on 56 samples collected from intensive care units (ICUs) and found biofilms on all samples (56/56) (Costa et al., 2019). Among other multi-drug resistant pathogens found on surfaces in ICUs, extended spectrum beta-lactamase producing *Klebsiella pneumoniae* and methicillin-resistant *Staphylococcus aureus* (MRSA) were found through conventional culturing techniques (Costa et al., 2019). Through SEM imaging, four hospital surfaces which tested positive for MRSA were found to be encased within biofilms. These studies present evidence about the predominant existence of bacteria in healthcare settings as biofilms. Coupled with findings that biofilms are on average 1,000 times harder to inactivate than free-floating bacteria and are responsible for approximately 75% of bacterial infections (Potera, 2010), these studies suggests that pathogens encased in biofilms continue to present challenges for the routine disinfection of environmental surfaces.

When biofilms are present on environmental surfaces, they can make surface hygiene more challenging. In healthcare facilities, this is especially a concern as there is a high-risk population with prolonged exposure to the environment. Here we note some general differences between biofilms relevant for this discussion. Biofilms are often divided into wet surface and dry surface biofilms, which is useful for discussing some macro level differences. Wet surface biofilms have a higher moisture level and can be from 300 to 500 um to several cm thick, often making them visible to the naked eye (Alfa, 2019). Dry surface biofilms have much less moisture and are typically 10–50 um in thickness, making them invisible to the naked eye in most situations (Alfa, 2019).

For healthcare facilities the issue of dry surface biofilms is a relatively recent concern. Starting with Vickery et al. (2012), Hu et al. (2015), Otter et al. (2015), Ledwoch et al. (2018), Tewes et al. (2022) finding that dry surface biofilms are commonly found (≥80%) on environmental surfaces and patient care equipment in healthcare facilities. Because dry surface biofilms are not visible to the naked eye, high resolution microscopy was needed to demonstrate their presence (Vickery et al., 2012). In a review article, Alfa discussed that dry surface biofilms likely play a role in healthcare associated infections (HAIs) as an environmental reservoir, but this is not well investigated to date (Alfa, 2019).

Because the biofilm matrix provides protection to bacteria and fungi within the matrix, there are questions around the ease with which hand contact with the biofilm can transfer bacteria to other surfaces or a patient. Tahir et al. (2019) demonstrated that *in vitro* bacterial biofilms could be readily transferred through touching coupons with gloved hands for all glove types tested. While tested under laboratory conditions rather than through real world testing, this study nonetheless demonstrated the potential for transmission of bacteria that are part of a dry surface biofilm.

Although biofilms are the preferred mode of microbial life, the detection of bacteria and fungi in biofilms can be challenging. Costa et al. (2019), demonstrated that surfaces with dry surface biofilms which were tested via qPCR and microscopy were positive in 100% (57/57) of samples, while surface swabbing only was positive in 45.6% (25/57) of samples, demonstrating that traditional swabbing methods may not be consistently effective in removing microorganisms from a dry surface biofilm.

Biofilms in sinks and drains have been implicated in HAIs as well. A 2014 study by Walker found that water taps contaminated with *Pseudomonas aeruginosa* biofilms were the likely source of *Pseudomonas aeruginosa* infections in patients (Walker et al., 2014). A retrospective study in German hospitals across 552 ICUs found that when there were handwashing sinks in patient rooms in the ICU, they were associated with a 21% increased risk of HAIs. This was believed to occur when bacteria in a biofilm in the drain cross contaminated other surfaces via splashing (Fucini et al., 2023).

## Biofilms on food contact and non-food contact surfaces

Food processing environments are also prone to environmental biofilms. The likely prevalence of biofilms in food processing environments is enhanced by an abundance of moisture, the food matrix and a wide range of hydrophobic or hydrophilic surfaces for bacterial adherence. *In vitro* studies have demonstrated the growth of *Escherichia coli*, *Listeria monocytogenes*, *Salmonella enterica, Staphylococcus aureus,* and *Pseudomonas aeruginosa* biofilms on common materials (stainless steel, rubber, plastic, Teflon) used for designing food contact surfaces (Di Bonaventura et al., 2008; Chia et al., 2009; Dourou et al., 2011; Di Ciccio et al., 2015; Wang Jingjin et al., 2015). However, biofilm formation on food contact surfaces may vary by material type. In a 2009 study, Chia et al. found that Teflon was a better substrate for the formation of *Salmonella* biofilms than stainless steel and glass (Chia et al., 2009). The inherent ability for stainless steel to represent an unsuitable substrate for the “rapid” formation of biofilms may have informed the common use of 304 stainless steel for the construction of most surfaces used in food processing environments. In another study, Di Ciccio et al. (2015) isolated 67 *S. aureus* strains from foods and food environments and found that 56% (38/67) were good biofilm formers on food contact surface materials made of stainless steel or polystyrene. Moreover, Di Ciccio et al. (2015) found that biofilm formation on food contact surfaces by *S. aureus* was temperature dependent. Specifically, among the *S. aureus* strains isolated from food handlers, only one was a good biofilm former at 12°C compared to 24 strains at 37°C. In a similar study, Bonaventura et al. found that at 37°C *Listeria monocytogenes* was a better biofilm former on stainless steel and glass compared to polystyrene (Di Bonaventura et al., 2008). In a 2015 study, Wang et al. found that persistent *L. monocytogenes* isolates from 30 US retail delis demonstrated an enhanced surface adhesion ability; a critical step for the establishment of biofilms (Wang Jingjin et al., 2015). Similarly, Dourou et al. (2011) investigated the formation of *Escherichia coli* O157:H7 biofilms at 15°C-the average temperature of non-production hours in US meat processing facilities and at 4°C for cold storage and found the attachment of *E. coli* O157:H7 on food contact surfaces. Without adequate hygiene interventions, *E. coli* could persist on stainless steel surfaces for up to 3 months (Warnes et al., 2012). Similarly, *L. monocytogenes* could persist for months on food contact and non-food contact surfaces with persistence being explained by the presence of transferable genetic elements such as plasmids and transposons (Palaiodimou et al., 2021). Similar to healthcare environments, bacterial biofilms are prevalent on diverse material types used for food contact and non-food contact surfaces. These studies demonstrate that while biofilms may readily be formed on environmental surfaces, local conditions, such as the substrate material type and environmental temperature, can affect the establishment of biofilms. This is evident as these studies surfaces (Di Bonaventura et al., 2008; Chia et al., 2009; Dourou et al., 2011; Di Ciccio et al., 2015; Wang Jingjin et al., 2015) demonstrate the ability for both Gram-positive and Gram-negative bacteria to adhere and persists on different surface material types commonly found in food processing environments.

## Biofilm-forming abilities vary by species and strain

Although microorganisms predominantly exist in biofilms, not all bacteria strains are biofilm formers. Specifically, the presence of bacterial surface proteins such as protein A and bacterial structures that enhance attachment onto biotic and abiotic surfaces such as fibrils differentiate biofilm formers from non-biofilm formers. Scanning electron microscope images of surfaces colonized by the non-pathogenic *S. epidermidis* ATCC 12228 have suggested the lack of an extracellular polymeric matrix characteristic of good biofilm formers (Di Ciccio et al., 2015). In an evaluation of 92 clonally unrelated but clinically relevant isolates of *Acinetobacter baumannii,* Rodriguez-Bano et al. (2007) found that 63% (58/92) were biofilm formers and 47% (34/92) were non-biofilm formers. Additionally, Espinal et al. (2012) collected two biofilm forming strains and two non-biofilm formers from the 92 clinically relevant strains investigated by Rodriguez-Bano et al. and found that non-biofilm forming strains of *A. baumannii* could survive on hard nonporous surfaces for approximately 15 days compared to 36 days for biofilm formers.

Although considered a “model” biofilm former, strains of *Pseudomonas aeruginosa* differ in their abilities to form biofilms. However, biofilm forming strains of *P. aeruginosa* can entrap non biofilm forming strains within the extracellular polymeric substances they secrete (Deligianni et al., 2010); forming a community better suited to withstand diverse environmental stressors. In a study of multi-species biofilms, Liu et al. (2017) found that the growth of poor biofilm formers such as *Microbacterium oxydans* was enhanced by the presence of good biofilm formers such as *Xanthomonas retroflexus* and *Stenotrophomonas rhizophila* common in soils. In a similar study with bacterial strains collected from food contact surfaces from the dairy industry in Europe, Sadiq et al. (2023) reported a threefold increase in biofilm mass when non-biofilm formers (*S. rhizophila, Bacillus licheniformis* and *Calidifontibacter indicus*) were grown with *M. lacticum*-a strong mono-culture biofilm former. However, the addition of *B. cereus* to mixed-culture biofilms resulted in an overall reduction in the biofilm mass secreted by the group. Although strain-dependent, these studies suggests that the co-existence of biofilm and non-biofilm forming strains may enhance the persistence and virulence of non-biofilm formers.

## Environmental microbiome is constantly changing

Environmental surfaces in healthcare settings could be inhabited by diverse microbial species. In a 2017 study, Yano et al. evaluated the species diversity on surfaces in small (<100 beds), medium (100–500 beds) and large (>500 beds) hospitals in Japan (Yano et al., 2017). Yano et al. found that regardless of hospital size, sampled surfaces were “home” to over 500 microbial species spread across 20 phyla (Sadiq et al., 2023). Pereira da Fonseca et al. investigated the microbial burden on elevator buttons, employee timers, ATM machine keyboards, and multiple restroom surfaces in a hospital in Brazil and found over 2,800 bacterial genera belonging to 926 families (Pereira Da Fonseca et al., 2016). Among the species reported by Pereira da Fonseca et al., potentially pathogenic bacteria such as *Klebsiella pneumoniae, Staphylococcus aureus,* and *Salmonella enterica* were reported. A similar study by Shobo et al. employed 16 s high throughput metagenomic techniques to evaluate 150 samples collected from surfaces in three public hospitals in South Africa. Shobo et al. (2020) identified 288 known species belonging to 190 genera. In an evaluation of the bacterial diversity of surfaces in two neonatal ICUs in San Diego, California, USA, Hewitt et al. found a mean of 93 genera per sampled surface (Hewitt et al., 2013). Although the microbial diversity found on healthcare surfaces may not be atypical, these studies highlight the need for well-tested routine disinfection regimes for surfaces in healthcare settings. The desire to selectively eliminate pathogens on environmental surfaces while “conserving” good members of the community which do not cause disease has been expressed (Shmidtchen et al., 2011; Li et al., 2016; Fakhrullina et al., 2019). However, to the best of our findings, routine hygiene interventions have not advanced to the level where “good” bacteria are preserved during the deactivation of pathogens.

The presence of pathogens on healthcare surfaces have been associated with the transmission of a wide range of bacteria including methicillin-related *Staphylococcus aureus, Klebsiella pneumonia, Acinetobacter baumannii,* Vancomycin resistant *Enterococcus, Eschericia coli, Pseudomonas aeruginosa,* and *Clostridioides difficile.* These surfaces have also been linked with the transfer of pathogens to the hands of healthcare personnel. Specifically, the survival of norovirus, *Clostridioides difficile* and *Acinetobacter* species on hospital surfaces have been demonstrated to transfer to the hands of healthcare workers (Weber et al., 2010). Additionally, the risk of hand contamination by *S. aureus* biofilms has been demonstrated; suggesting that the biofilm mode of life does not limit bacteria transfer to the hands of healthcare personnel (Chowdhury et al., 2018).

The microbial diversity of most environments may however be dynamic. Seasonal, temporal, and spatial variations in the microbiome of the air, hard non-porous surfaces and soft porous surfaces (such as bedsheets) in healthcare settings have been investigated. In a Varshney et al. study, polyester-cotton (70:30 blend) fabrics were stitched on bedsheets used by patients and microbial loads evaluated for 7 months through qPCR and 16S rRNA amplicon sequencing. Varshney et al. (2022) found seasonal variations in bacterial densities on patient bedsheets with maximum loads reported on the second of the seven-month study. Although patient rooms are typically temperature-controlled, Varshney et al. (2022) found a positive correlation between outside temperature and variations in bacterial loads on patient bedsheets. Specifically, when outside temperature and relative humidity were lowest, bacterial loads on patient bedsheets were the least during the study period (Varshney et al., 2022). This may suggest the need for season-specific standard operation procedures for hospital laundry. Moreover, a quarterly comparison of the alpha diversity of patient bedsheets revealed significant differences (*p* < 0.0001) in the relative abundance of microorganisms with seasonal changes (Varshney et al., 2022). Although *S. aureus, Klebsiella pneumoniae* and *Escherichia-Shigella* were detected on bedsheets throughout the study period, the same was not true for the ubiquitous *P. aeruginosa* (Varshney et al., 2022). The seasonal variability of microbial species on environmental surfaces may further challenge efforts to develop selective antimicrobials which only target the deactivation of pathogens regardless of them being in the planktonic state or in biofilms.

In an evaluation of the microbiome of 36 porous and 36 non-porous surfaces in an outpatient REHAB clinic, Brigando et al. (2023) found that surface material type significantly impacted the bacterial load on sampled surfaces. The same group found that within a healthcare facility, porous surfaces were more contaminated by bacteria than non-porous surfaces probably owing to the presence of multiple “crevices” serving as hiding sites on non-porous surfaces. Bacterial and fungal population changes on the porous and non-porous surfaces investigated by Brigando et al. were however, not impacted by the frequency of contact; suggesting that routinely touching healthcare surfaces may not significantly impact changes in the microbiome of high touch surfaces. This was further substantiated when Brigando et al. found no significant difference in the mean bacterial loads on high and low touch surfaces. Additionally, Shannon diversity indexes were similar for high and low touch surfaces; non-porous and porous surfaces. Similarly, cleaning frequency did not impact the total bacterial load detected on porous and non-porous surfaces.

The standard intervention to improve surface hygiene for environmental surfaces and equipment is to clean and disinfect or sanitize the surface. Little is known about the impact on the microbiota from cleaning/sanitizing/disinfecting, but it seems logical that it would alter the microbiota of any surface. A US Center for Disease Control funded study by Perry-Dow found that using 16S rRNA sequencing they were able to show changes in the microbiome of the surfaces in a patient room and that the microbiome was different for rooms where sodium hypochlorite disinfectant was used when compared to rooms where a quaternary ammonium chloride disinfectant was used Perry-Dow et al. (2022). Rooms disinfected with sodium hypochlorite had higher levels of Gram-positive species while rooms disinfected with a quaternary ammonium chloride disinfectant had higher levels of Gram-negative species. This suggests that while changes in microbial populations on healthcare environments could be seasonal or influenced by floor traffic and the skin microbiota of patients, chemical hygiene could also play a major role in changing the population dynamics of pathogens on healthcare surfaces.

## Gene transfer and expression in biofilms

Within every community of microorganisms, genetic material can be transferred in one or more of several ways. From parents to progenies, vertical gene transfer occurs. Among usually unrelated microbial species, lateral gene transfer allows for the exchange of genetic material generally referred to as mobile genetic elements (MGEs). MGEs may carry virulent traits such as genes encoding for antimicrobial resistance and biofilm formation; highlighting the critical role MGEs may play in multi-species biofilms (Madsen et al., 2012; Ma et al., 2021).

Multi-species biofilms have a dense and tightly-packed population structure. Among others, this structure is maintained by conjugation pili which may serve as cell adhesins; connecting cells together in a tighter structure (Madsen et al., 2012). This inter-connectedness among multiple species is important for the formation of micro-colonies; a critical stage in the formation of mature biofilms. Compared to planktonic cells, higher conjugation frequencies are observed in biofilms (Hausner and Wuertz, 1999). In multi-species biofilms, this may enhance the transfer of mobile genetic elements (MGEs), such as plasmids (Ma et al., 2021) as Warnes et al. (2012) found that higher conjugation frequencies were observed among *Escherichia coli* strains when donor strains carried horizontally acquired genes. In an earlier study, Hausner and Wuertz (1999) found that at the center of multi-species biofilms, the population of MGE recipients were approximately two times the number of MGE donors. In a later study, Warnes et al. (2012) found that the horizontal acquisition of β-lactamase genes by azide-resistant *Escherichia coli* strains on stainless steel surfaces was mediated by plasmids when plasmid recipients and donors co-existed on the same surface. These studies suggests that the exchange of MGEs plays an important role in sustaining the persistence and co-existence of MGE recipients and donors on abiotic surfaces.

MGEs could also allow for the transfer of antimicrobial resistance genes among bacteria encased in biofilms as Ma et al. (2021) found that in *Campylobacter jujeni* biofilms, the potential for antimicrobial resistance genes to be transferred laterally increased by 17-fold compared to their planktonic counterparts. This suggests that the biofilm way of life may enhance the transfer and acquisition of antimicrobial resistance genes and in so doing, continue to challenge chemical hygiene efforts. Although antimicrobial resistance may be a concern, this is usually the result of repeated exposure to sub-lethal doses. However, typical use concentrations for commercially available disinfectants are orders of magnitude above the minimum biocidal concentrations required to irreversibly inactivate pathogens from environmental surfaces; possibly reducing chances for antimicrobial resistance build up.

Unlike vertical gene transfer, lateral gene transfer could result in nouvelle genetic combinations in biofilms (Madsen et al., 2012). A lateral transfer of mobile genetic elements may facilitate the persistence of molecular parasites. However, the transfer and persistence of MGEs within biofilms is dependent on nutrient availability (Madsen et al., 2012). In the oxic zone of biofilms, lateral gene transfer frequencies are higher compared to suboxic and anoxic regions of the same biofilm (Madsen et al., 2012). This suggests that the thickness of a biofilm may impact the persistence of pathogens on environmental surfaces.

Gene transfer through MGEs may not necessarily translate into gene expression. In a study of the genes expressed in biofilms, Ruiz-Sorribas et al. (2021) investigated the expression of virulence genes in dual-and three-species biofilms of *S. aureus* ATCC 25923, *Candida albicans* ATCC 24433, and *E. coli* ATCC 47076. The Ruiz-Sorribas group found an increased expression of *S. aureus* virulence genes such as haemolysin alpha (*hla*) in multi-species biofilms with hyphae-producing *C. albicans* (Ruiz-Sorribas et al., 2021). Similarly, the expression of PNAG synthase (icaA) known to significantly contribute to the synthesis of exopolysaccharides in *S. aureus* biofilms was upregulated in 28 to 48 h old biofilms of *S. aureus* and *C. albicans* (Ruiz-Sorribas et al., 2021). Specifically, in these biofilms, there was a strong correlation between hyphae-rich *C. albicans* biofilms and the expression of PNAG synthase in *S. aureus*; suggesting the exchange of mobile genetic elements between both species. Moreover, in inter-kingdom biofilms of *C. albicans* and *S. aureus,* the expression of phenol-soluble modulin alpha (*psmα*) responsible for the dispersion of *S. aureus* was also up-regulated in 48 h old biofilms. This may be indicative of an increased colonization risk of otherwise “clean” surfaces by planktonic cells with enhanced virulence traits. This in addition to the presence of efflux pumps, persister cells and the secretion of extracellular polymeric substances may contribute to making biofilms up to 1,000 times harder to inactivate than planktonic cells.

## Implications of mobile genetic elements for surface disinfection

The pan-genome of microorganisms is made of the core genome and the non-core genome (Rankin et al., 2011). While the core genome is typically transferred vertically, the non-core genome is transferred horizontally. The non-core genome contains non-expressed genes, recently acquired traits and mobile genetic elements (MGEs). MGEs carry multiple genetic elements such as plasmids and bacteriophages and are largely responsible for horizontal gene transfer. Horizontal gene transfer through MGEs could involve the uptake of naked DNA or the transfer of bacteriophages as vectors with foreign DNA (Rankin et al., 2011). This suggests that antimicrobials with the innate ability to target and destroy nucleic acids may be better suited to reduce the burden of pathogens that have acquired virulence traits horizontally.

Plasmids transferred horizontally could carry multiple virulence genes. In *Escherichia coli* O157:H7, the pO157 plasmid carries *CAT* genes that encode for the expression of catalases known to breakdown hydrogen peroxides into water and oxygen (Pan et al., 2022). Additionally, biofilm associated proteins (Bap) may be contained in MGEs. This suggests that some bacterial species may have acquired antimicrobial tolerance genes through MGEs; enhancing their tolerance to sub-lethal antimicrobial concentrations. In the presence of sub-lethal doses (4 ppm) of quaternary ammonium compounds (QAC), Casey et al. (2014) found a minimum four-fold increase in the expression of *L. monocytogenes* virulence genes. Compared to zero exposure to QAC, exposure to sub-lethal doses of QAC triggered an upregulation of carbohydrate uptake, bacterial chemotaxis and peptidoglycan biosynthesis in *L. monocytogenes* (Casey et al., 2014). The increased biosynthesis of peptidoglycans in the presence of sub-lethal QAC concentrations suggests a thickening of the Gram-positive bacterial cell wall. This may present particular sanitization challenges as the QAC mode of action depends on a lysis of the bacterial cell membrane. Lipus et al. (2019) exposed *Pseudomonas fluorescence* biofilms to sub-lethal concentrations of sodium hypochlorite (0.6 ppm) and found an increased transcription of genes associated with oxidative stress repair, multi-drug efflux pumps and peroxide scavenging enzymes such as alkyl hydroperoxide reductase. These studies reiterate the need for end-users of antimicrobial products to strictly adhere to label use conditions. Label use conditions for antimicrobials are typically orders of magnitude above sub-lethal and minimum inhibitory concentrations; allowing innate defense mechanisms that are up-regulated following exposure to sub-lethal concentrations to be overwhelmed.

The transfer of MGEs promotes collaboration among bacteria (Dimitriu et al., 2014). Bacterial collaborations could be exhibited by the widespread existence of multi-species biofilms in nature. Specifically, biofilm associated proteins (Bap) known to enhance biofilm formation could be transferred among microbial species through MGEs (Lasa and Penadés, 2006); allowing non-biofilm formers to acquire biofilm-forming abilities. MGEs are incorporated into ubiquitous yet highly conserved chromosomal sequences that participate in horizontal and vertical gene transfers. However, horizontally acquired genes can be transferred vertically during microbial growth and reproduction; improving their spread and ensuring the persistence of pathogens of concern on environmental surfaces.

Cohen et al. (2018) performed a sub analysis on the antibiogram of *Klebsiella pneumoniae* clinical samples of paired patients, where the initial patient had a *K. pneumoniae* infection and the subsequent patient in the same room acquired a *K. pneumoniae* infection, classified as HAIs. They found that for 38 case-pairs, 58% (22/38) had identical antibiograms, 29% (11/38) displayed additional antibiotic resistance for the second patient, and 13% (5/38) showed reduced antibiotic resistance for the second patient. When the antibiotic resistance of Klebsiella changed, the bacteria was roughly twice as likely to acquire additional antibiotic resistance as to become less antibiotic resistant. This data suggests that acquisition of additional antibiotic resistance can be associated with exposure to the healthcare environment. Whole genome sequencing was not performed in this study, which would help demonstrate the genetic relatedness of the isolates.

## Disinfectant efficacy against biofilms versus planktonic cells

While it is well established that disinfectants can effectively kill or inactivate pathogenic microorganisms on surfaces, microorganisms can be less susceptible to disinfectants when in an environmental biofilm (Maillard and Centeleghe, 2023). This “resistance” to disinfection may increase as biofilms age and secrete thicker and more compact layers of extracellular polymeric substances.

Several studies have investigated the efficacy of disinfectants against biofilms under various sets of conditions. Machado demonstrated that when *Pseudomonas aeruginosa* and *Escherichia coli* biofilms were exposed to low levels of benzalkonium chloride, this induced an adaptive response from the bacteria, increasing the mass of the biofilm (Machado et al., 2012). Almatroudi et al. (2016) found that while exposure of a dry surface biofilm of *Staphylococcus aureus* to 1,000 ppm to 20,000 ppm of sodium hypochlorite initially reduced the biofilm biomass, live cells remained and regrew during incubation for all tested levels of sodium hypochlorite. Tiwari et al. found that when sodium hypochlorite and 70% ethanol were compared for their efficacy against clinical isolates of *Staphylococcus aureus* vegetative cells and in a biofilm, 70% alcohol consistently has less efficacy than the sodium hypochlorite solutions despite both being commonly used for surface disinfection (Tiwari et al., 2018). Similarly, Ledwoch et al. (2021) found that for 11 disinfectants tested for their ability to reduce bacteria levels in a dry surface biofilm in a lab study, only two completely removed the dry surface biofilm with eight of the other nine regrowth occurring within 2 days.

Lineback et al. (2018) tested a range of registered disinfectants against wet surface biofilms of *Staphylococcus aureus* and *Pseudomonas aeruginosa* using the standard EPA test method for wet surface biofilms and found that the two tested quaternary ammonium chloride products containing disinfectants failed to achieve the minimum six log reduction required by the US Environmental Protection Agency. Chaggar et al. tested a range of registered disinfectants against dry surface biofilms using the method developed by Nkemngong et al. (2020) and similarly found that quaternary ammonium chloride containing disinfectants failed to perform as well as the sodium hypochlorite and hydrogen peroxide disinfectants (Chaggar et al., 2022). These studies suggest a general weakness for quaternary ammonium chloride containing disinfectants to kill bacteria in biofilms.

Current disinfectant registration processes in the United States and European Union require efficacy testing using standardized methods. The required methods in both regions mandate the use of microbiological testing methods that use planktonic organisms, including for disinfectants intended for use in healthcare facilities. While in the US the Environmental Protection Agency (EPA) has approved efficacy testing methods for wet surface biofilms against *S. aureus* and *P. aeruginosa* (ASTM E2562-12/ASTM E2871-12), these methods are optional for the registrant unless the registrant wants to make claims against wet surface biofilms (US Environmental Protection Agency office of Pesticide Programs. EPA, 2022). However, although dry surface biofilms are prevalent and present higher disinfection challenges, the EPA currently has no approved efficacy methods for making dry surface biofilm claims, although at least one has been proposed based on dehydration of biofilms made using the EPA approved wet biofilm method (Nkemngong et al., 2020). In Europe, the European Committee for Standardization (CEN) does not currently have approved efficacy methods for wet surface or dry surface biofilms for hard surface disinfectants (European Committee for Standardization, 2023). Consequently, healthcare facilities are not generally able to know whether the disinfectants and sanitizers used in their environmental hygiene program are effective against biofilms and the embedded microorganisms unless the manufacturer has run additional testing against biofilms.

## Conclusion

Although prevalent on environmental surfaces, the impact of environmental biofilms on the risk of healthcare associated infections is poorly understood. Despite the wealth of scientific evidence that exists on the prevalence of biofilms on environmental surfaces, carrying a biofilm claim on an antimicrobial product label still remains optional. We recommend that to adequately mitigate infection risks linked to environmental surfaces, antimicrobials with demonstrable efficacies against mixed culture and inter-kingdom biofilms may be more reliable. Additionally, to ensure *in vitro* biofilm assays are reflective of the biochemical complexities of real-world biofilms, tests that promote the exchange of mobile genetic elements among pathogens of interest may be relevant for irreversibly inactivating biofilms.

## Author contributions

CN: Writing – review & editing, Writing – original draft, Project administration, Conceptualization. PT: Writing – review & editing, Writing – original draft, Conceptualization.
